# Epidemics of viral meningitis caused by echovirus 6 and 30 in Korea in 2008

**DOI:** 10.1186/1743-422X-9-38

**Published:** 2012-02-15

**Authors:** Hye-Jin Kim, Byounghak Kang, Seoyeon Hwang, Jiyoung Hong, Kisang Kim, Doo-Sung Cheon

**Affiliations:** 1 Division of Enteric and Hepatitis Viruses, Center for Infectious Diseases, National Institute of Health, Korea Center for Disease Control and Prevention, Chungcheongbuk-do 363-951, South Korea; 2 Department of Biology, College of Sciences, Kyung Hee University, Seoul, Korea

**Keywords:** Enteroviruses, Echovirus 30, Echovirus 6, Aseptic meningitis

## Abstract

**Background:**

Enteroviruses (EVs) are the leading cause of aseptic meningitis, which is the most frequent central nervous system infection worldwide. We aimed to characterize the EVs involved in an aseptic meningitis outbreak in Korea in 2008. In Korea, Echovirus type 30 (E30) and E6 have been associated with outbreaks and frequent meningitis.

**Methods:**

During 2008, through nationwide surveillance, we collected specimens from 758 patients with aseptic meningitis-related clinical manifestations. The detection of EVs from specimens was subjected to a diagnostic real-time RT-PCR in the 5' NCR. A semi-nested polymerase chain reaction (PCR) to amplify sequences from the VP1 region and sequence comparison with reference strains registered in Genbank was performed for the genotype determination.

**Results:**

Most patients (98%) in this outbreak were children < 15 years of age. The temporal distribution of the E6 and E30 epidemics showed an obvious seasonal pattern during the short period from June to July. A large majority of the EV-positive patients experienced fever, headache, vomiting, and neck stiffness. Some patients also showed cold symptoms, sore throat, altered mental status, and seizures. We did not observe a higher fatality rate in children with E6 or E30 infection. Most of the patients recovered uneventfully. In most cases, the cerebrospinal fluid (CSF) profile was studied, and generally showed a higher than normal white blood cell count (≥ 5/mm^3^). We detected EVs from 513 patients (67.68%) and identified the EV genotype in 287 patients. E30 (n = 155, 50.4%) and E6 (n = 95, 33.1%) were the predominant genotypes. E9, E1, E7, E16, coxsackievirus A3, 4, 6, coxsackievirus B1, 3, and 10 were also identified. According to phylogenetic analysis, E30 belonged to subgroup 4b, and E6, to the C4 subgroup.

**Conclusions:**

Conclusively, aseptic meningitis was the most common manifestation in children with either echovirus 30 or 6 infection. Identification of E6 and E30 as the prominent EVs in the 2008 outbreak in South Korea shows the potential of EVs to cause a serious disease in an unpredictable (fashion. Our findings provide new) insights into the clinical and virological features of the aseptic meningitis outbreak caused by E30 and E6.

## Background

Enteroviruses (EVs) are the leading cause of aseptic meningitis, which is the most frequent central nervous system infection worldwide. Aseptic meningitis in children is the most commonly encountered serious illness associated with enteroviral infections, often appearing in the form of an outbreak [1-3]. EVs are classified into 4 species: (1) *Human enterovirus A *(coxsackievirus [CV] A2-A8, A10, A12, A14, and A16; EV71, 76, 89, 90, and 91); (2) *Human enterovirus B *(CVA9 and CVB1-CVB6; echovirus 1-7, 9, 11-27, and 29-33; EV69, 73-75, 77-88, 97, 100, and 101); (3) *Human enterovirus C *(CVA1, A11, A13, A17, A19-A22, and A24; polioviruses 1-3; EV96); and (4) *Human enterovirus D *(EV68 and EV70) [4].

Echovirus (E) is the major causative agent of aseptic meningitis. E6, E9, E11, E13, E19, and E30 are the most common EV genotypes detected from patients with aseptic meningitis, either in an epidemic or endemic form of the disease [5-9]. Since the development of reverse transcription-polymerase chain reaction (RT-PCR) assays for the detection and molecular typing of EVs from clinical samples, many aseptic meningitis outbreaks have been described worldwide during the last few years [10,11]. E30 is one of the most frequently detected EVs in the United States, accounting for 10.1% of all reported EVs detected from 1970 to 2005; E6 accounted for 6.2% [12]. In Korea, nationwide surveillance was initiated in 1993 and a number of summer outbreaks caused by various EVs, including E6, E9, E13, E30, and CV A24 has been reported [13].

The present study aimed to detect and characterize the EVs associated with an aseptic meningitis outbreak in Korea in 2008. In Korea, E6 and E30 have been associated with outbreaks and frequent meningitis. We performed clinical and etiological investigations of EV infections through a nationwide surveillance.

## Methods

### Patients and epidemiological investigation

A nationwide surveillance system for EV infections was initiated in Korea in 1993. Twenty-nine clinics managed by experienced pediatric physicians participated in 2008. The physicians sent specimens collected from patients in whom the viral disease was highly suspected, and reported the demographic characteristics and clinical symptoms of the patients. We collected 954 specimens from 758 patients who presented with aseptic meningitis in 2008. The participating clinics provided cerebrospinal fluid (CSF), stool or rectal swab, throat swab, serum and urine samples of the patients in whom the viral disease was suspected. Meningitis is defined pleocytosis as a white blood cell (WBC) count of ≥ 5/mm^3 ^in the CSF, substantial meningeal enhancement as identified by brain computed tomography or magnetic resonance imaging, and/or neurological dysfunction as defined by the physician.

During 2008, samples from aseptic meningitis patients were sent to the Korea Center for Disease Control. The patients' samples were confirmed as EV-positive, and information regarding age, sex, and duration of illness was recorded. To elucidate the relationship between E30 and E6 isolates of diverse temporal and geographic origin, the partial VP1 sequences of the E30 and E6 outbreak isolates were compared with each other, and with those of E30 and E6 isolates in our collection.

### Enterovirus detection and molecular typing

The specimen from patient including stool, CSF, throat swab and so on were used for the EV detection and molecular typing. Viral RNA was extracted using magnetic beads(GM- AUTOPREP™ Kit, Korea), and the purified viral nucleic acid was processed using Freedom EVO (Tecan, USA). EV detection was processed in the one step real-time RT-PCR. We performed using a dual-labeled fluorogenic EV-specific probe and primer a high conserve 5'- noncoding region as the target of a 196 bp region described by Verstrepen *et al*. [14]. For molecular typing and phylogenetic analysis, the VP1 amplicons generated by semi-nested RT-PCR were sequenced and found to correspond to a 372-bp region of the VP1 gene followed by US, Center for Disease Control. For subgenotype confirmation of the virus, we used the capsid region of the VP1 fragment as a target for a semi-nested RT-PCR, followed by sequencing. To determine the EV type, we determined the sequence homology between the amplified EV PCR products and the VP1 sequences available in GenBank.

### Sequence and phylogenetic analysis

The sequences obtained were identified in terms of closest homology by using BLAST http://blast.ncbi.nlm.nih.gov/Blast.cgi. Multiple sequence alignments with the respective reference strain sequences were made using BioEdit Sequence Alignment Editor. MEGA software (version 4.0) was used for phylogenetic analysis. Phylogenetic trees were constructed using the neighbor-joining method (bootstrap resampling of 1000 replicates). Sequence data from both strands were aligned and edited using MEGA. The partial VP1 sequences of KOR08-109, KOR08-132, KOR08-151, KOR08-338, KOR08-518, KOR08-ES42, KOR08-ES138, KOR08-ES157, KOR08-ES168, KOR08-ES432, and KOR08-ES481 have been deposited in GenBank with the accession numbers HQ833325-HQ833329 and JF267786-JF267791.

## Results

### Epidemiological features of enteroviral aseptic meningitis

An outbreak of aseptic meningitis affected 758 patients in South Korea in 2008. Most patients (98%) in this outbreak were children < 15 years of age. The sex distribution showed that 463 (61.1%) patients were male, and 269 (35.5%) were female. Information regarding the sex of 26 patients was unavailable.

**Figure 1:**
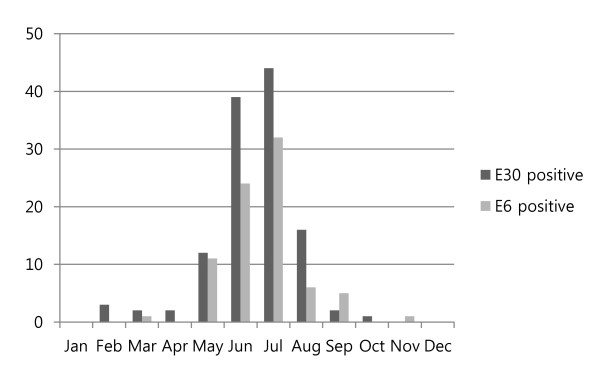
**Seasonal distribution of E6 and E30 in patients with aseptic meningitis in Korea in 2008**.

The temporal distribution of the epidemics showed an obvious seasonal pattern and occurred during the short period from June to July. E30 and E6 were most frequently detected from the EV-positive patients (Figure 1).

Specimens classified CSF, stool, throat swab, serum and urine samples in the different type from 758 patients. At the 613 specimens of the 513 patients detected enterovirus as a cause of aseptic meningitis. Our study has a large number of CSF (n = 443, 46.4%), nevertheless the positive rate was the highest in the stool (77.1%) (Table 1).

**Table 1:** The source of specimens(n = 613) for enterovirus-positive identified from 758 patients presented with aseptic meningitis

Type of specimens	Positive(n = 613)	positive rates	Total(= 954)	Proportion
Stool	299	77.1%	388	40.7%

CSF	262	59.1%	443	46.4%

Throat swab	28	50.9%	55	5.8%

urine	21	45.7%	46	4.8%

Serum	3	13.6%	22	2.3%

### Clinical features of enteroviral aseptic meningitis

The clinical features of the patients with aseptic meningitis infections are shown in Table 2. A large majority of the EV-positive patients experienced fever, headache, vomiting, neck stiffness, and occasional abdominal pain. Symptoms of fever, headache, and vomiting were more frequently observed in patients with E6 infection (>;10%) than in those with E30 infection. Some patients also showed signs of altered mental status, seizures, cold symptoms, and sore throat. Abdominal pain was more frequently observed in patients with E6 infection (n = 13) than in those with E30 infection (n = 9).

**Table 2:** The symptoms of enterovirus-positive patients with aseptic meningitis in Korea in 2008

	EV- positive	EV- positive rate (%)	E30	E30- positive rate (%)	E6	E6- positive rate (%)
Fever (> 37.3°C)	386	75.2	108	69.7	78	82.1

Headache	381	74.3	107	69.0	82	86.3

Vomiting	343	66.9	92	59.4	82	86.3

Neck stiffness	120	23.4	35	22.6	30	31.6

Erythematous rash	1	0.2	0	0.0	0	0.0

Vesicular rash	1	0.2	1	0.6	0	0.0

Sore throat	12	2.3	4	2.6	4	4.2

Cold symptom	7	1.4	2	1.3	2	2.1

Eye discharge	0	0.0	0	0.0	0	0.0

Eyeball pain	0	0.0	0	0.0	0	0.0

Myalgia	3	0.6	1	0.6	0	0.0

Abdominal pain	35	6.8	9	5.8	13	13.7

Diarrhea	4	0.8	1	0.6	1	1.1

Altered mental status	1	0.2	0	0.0	0	0.0

Seizure	2	0.4	0	0.0	0	0.0

Photophobia	1	0.2	0	0.0	1	1.1

Paralysis/Weakness	0	0.0	0	0.0	0	0.0

In 468 patients with meningitis, the CSF profile showed a higher than normal WBC count (≥ 5/mm^3^), whereas in 21 patients, the WBC count was < 5/mm^3^. This information was not available for 24 cases. Of the 331 patients who were EV positive, 6 had a WBC count < 5/mm^3^. Fifty-three patients positive for E6 and 95 positive for E30 had a WBC count ≥ 5/mm^3^.

### Enterovirus detection and molecular typing

**Figure 2:**
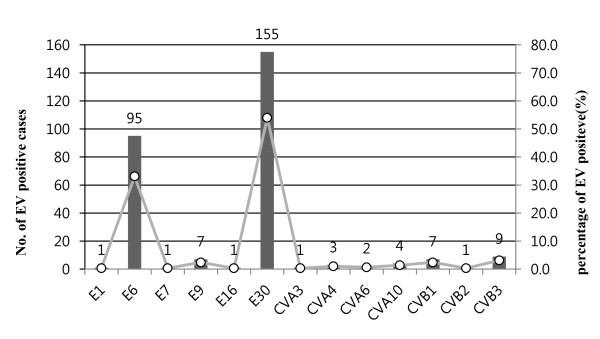
**The genotype distribution of enteroviruses identified in patients with aseptic meningitis in Korea in 2008**. Bar graph showing the number of enterovirus genotype. Open circles, genotype proportion of entire enterovirus.

The EVs detected from 758 patients with aseptic meningitis and other EV-related diseases were subjected to a diagnostic real-time RT-PCR in the 5' NCR. We successfully detected EVs from 513 patients (68%) with aseptic meningitis, and identified the EV genotype in 287 patients (56%). For molecular typing and phylogenetic analysis, the VP1 amplicons generated in the semi-nested RT-PCR were sequenced and found to correspond to a 372-bp region of the VP1 gene. The 287 amplicons were genotyped as E30 (n = 155, 50.4%); E6 (n = 95, 33.1%); E9 (n = 7, 2.4%); E1, E7, E16 (n = 1 each, 0.3%); CB3 (n = 9, 3.1%); CB1 (n = 7, 2.4%); CA10 (n = 4, 1.4%); CA4 (n = 3, 1.0%); CA6 (n = 2, 0.7%); and CA3 (n = 1, 0.3%). The other 226 samples did not generate VP1 amplicons in the semi-nested PCR and were therefore considered untypable (Figure 2).

### Sequence comparison and phylogenetic analysis of E6 and E30

We identified the EV genotype in 287 patients with aseptic meningitis. The distribution of the detected EV genotypes is shown in Figure 2. E30 (30.2%) and E6 (18.5%) were the predominant genotypes detected.

**Figure 3:**
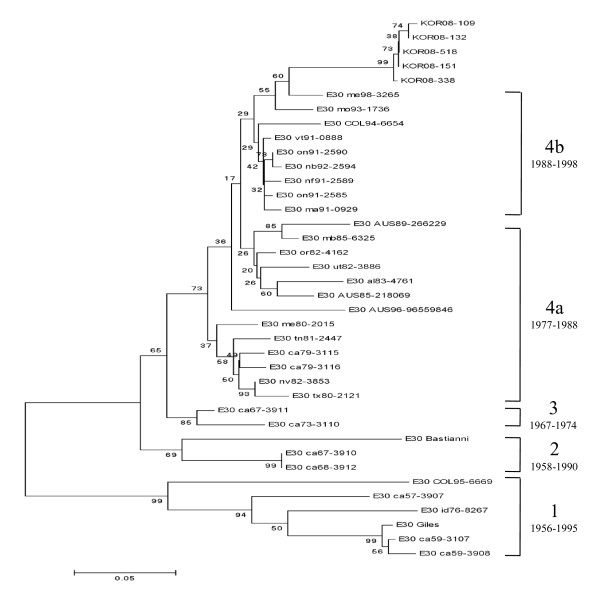
**Phylogram based on the alignment of the E30 VP1 gene nucleotide sequences**. Phylogenetic tree based on the alignment of the VP1 nucleotide sequences showing genetic affinity of the E30 samples from the 2008 outbreak in Korea. The neighbor-joining method with a maximum likelihood distance matrix was used to construct the tree. Numbers at the nodes represent the percentage of 1000 bootstrap pseudoreplicates.

**Figure 4:**
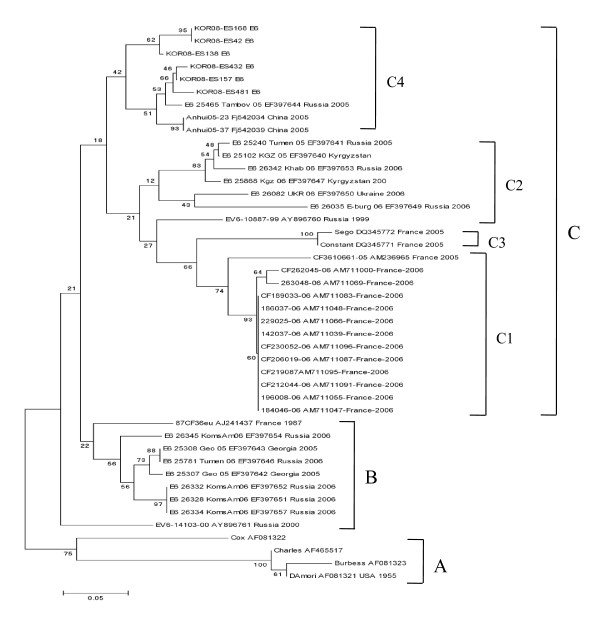
**Phylogram based on the alignment of the E6 VP1 gene nucleotide sequences**. Phylogenetic tree based on the alignment of partial VP1 nucleotide sequences of the Korean isolates and other strains of E6 of different geographic origins. The neighbor-joining method with a maximum likelihood distance matrix was used to construct the tree. Numbers at the nodes represent the percentage of 1000 bootstrap pseudoreplicates.

Three E6 genetic groups designated A, B, and C (subgroups C1, C2, C3, and C4), and 4 E30 genetic groups designated 1, 2, 3, and 4 (subgroups 4a and 4b) have been identified [15,16]. Based on this classification, the E30 detected in the present study belonged to subgroup 4b (Figure 3), and the E6 belonged to subgroup C4 (Figure 4).

## Discussion

In this study, we assessed the clinical and virological features of an aseptic meningitis outbreak caused by E30 and E6 in South Korea during the summer of 2008. E30 has been reported to cause frequent outbreaks of aseptic meningitis [15,16]. In Korea, aseptic meningitis outbreaks were caused by E6 and E30 in 2002 and 2006, respectively. E30 and E6 emerged as the predominant EV genotypes in 2008, and were associated with numerous outbreaks and significant morbidity.

Meningitis outbreaks typically occur in restricted geographical areas or communities during the summer and autumn seasons and lead to increased hospital admissions for short periods [16,17]. However, a few winter outbreaks of meningitis caused by EVs have been reported [18]. The distribution of E6 and E30 epidemics in Korea showed an obvious seasonal pattern during the short period from June to July. Stool or CSF specimens were the two most common sources of the specimens with enterovirus in our study. About reason for a large number of CSF specimen (n = 443, 46.4%) by comparison with other specimens, aseptic meningitis is the most common central nervous system infection in which bacterial agents cannot be identified in the CSF, and is characterized by the acute onset of signs and symptoms of meningeal inflammation [2]. The sensitivity of specimens from stool (77.1%) was higher than for CSF (59.1%) (Table 1). CSF from the patient suspected with aseptic meningitis induced enterovirus infection but the specimen with the highest sensitivity for establishing an acute infection is a stool specimen, regardless of clinical presentation [19]. Enterovirus genome detection or virus isolation in aseptic meningitis, stool specimen or throat swab is preferable for the diagnosis due to false negative induced from short term appearance of enterovirus and lower viral titers in the CSF specimen. Stool specimen or throat swab can be good diagnostic specimen for the detection of enteroviruses in the patients if we have clinical information compatible with viral meningitis based on practical view. In most cases in the present study, including 468 meningitis cases and 331 EV-positive cases, the CSF pleocytosis profile showed a higher than normal WBC count (≥ 5/mm^3^). Twenty-one EV-positive patients had a WBC count < 5/mm^3^.

The typical presentation of enteroviral meningitis in children is vomiting, anorexia, rash or respiratory symptoms, and meningism, often preceded by flu-like symptoms and sore throat [20]. Most patients who were positive for EV experienced headache, fever, vomiting, neck stiffness, and occasional abdominal pain. Some patients also exhibited symptoms of cold, altered mental status, and seizures. In addition to aseptic meningitis, E6 and E9 infections have been reported to be associated with encephalitis, rashes, and gastrointestinal illnesses [12], which were relatively uncommon in the present study. Fever, headache, vomiting, and neck stiffness were identified in > 20% of the patients. The US surveillance data indicate that E6 is a relatively virulent genotype with the highest fatality rate (5.6%) among all echoviruses. In the present study, we did not observe a higher fatality rate in children with E6 infection. Most meningitis patients with echovirus infections recovered uneventfully, although fatal case occurred in rare instances.

We detected EVs from 513 (67.68%) of the 758 aseptic meningitis patients in 2008. E30 (n = 155, 54%) and E6 (n = 95, 33.1%) were the predominant genotypes detected. E1, E7, E9, E16, CB3, CB1, CA10, CA4, CA6, and CA3 were also detected. In 2008, E30 presented a remarkably close group with 100% bootstrap value, and belonged to subgroup 4b. E30 is known to cause outbreaks by maintaining its isolation rates and increasing its circulation periodically [11,21]. E30 has been shown to undergo genetic variation over time, and this variability has been associated with changing circulation. In the present investigation of an E30-associated aseptic meningitis outbreak in Korea, the prevalent genotype was type 4b. All the 2008 strains of E30 and/or E6 detected in Korea exhibited 98-100% identity at the nucleotide and belonged to 1 genotype. E6 genetic groups have been designated as A, B, and C (subgroups C1, C2, C3, and C4). Based on this classification, E6 belonged to subgroup C4 (Figure 4).

## Conclusions

Conclusively, aseptic meningitis was the most common manifestation in children with either echovirus 30 or 6 infection. The identification of E6 and E30 as the prominent EVs in the 2008 outbreak in South Korea shows the potential of EVs to cause serious disease in an unpredictable fashion. Our findings provide better insights into the clinical and virological features of the aseptic meningitis outbreak caused by E30 and E6.

## Abbreviations

EV: Enterovirus; E: Echovirus; CSF: Cerebrospinal fluid; CVA: Coxsackievirus A; CVB: Coxsackievirus B.

## Competing interests

The authors declare that they have no competing interests.

## Authors' contributions

SYH, JYH and HJK performed genome analysis and cell culture. HJK drafted the manuscripts. BHK and KSK contributed to collection specimen and clinical diagnosis. DSC designed the study and critically revised the manuscript. All of the authors read and approved the final version of the manuscript.
